# Immunogenicity and Protective Efficacy of Baculovirus-Expressed SARS-CoV-2 Envelope Protein in Mice as a Universal Vaccine Candidate

**DOI:** 10.3390/vaccines12090977

**Published:** 2024-08-28

**Authors:** Tuba Çiğdem Oğuzoğlu, Alireza Hanifehnezhad, Saber Delpasand Khabbazi, İlke Karayel-Hacıoğlu, Onur Kaynarcalıdan, Zehra Fırat, Nazlıcan Filazi, Eda Erdem-Şahinkesen, Buket Gül, Muhammed Cesim Karabulut, Enes Koba, Ece Adıgüzel, Elif İrem Şenlik, Emrah Korkulu, Cansu Demirden, İlker Şahinkesen, Ahmet Ceylan, Hacer Muratoğlu, Sevil Vural, Zihni Demirbağ, Aykut Özkul

**Affiliations:** 1 Department of Virology, Faculty of Veterinary Medicine, Ankara University, Ankara 06070, Türkiye; 2 Department of Agriculture and Food, Institute of Hemp Research, Yozgat Bozok University, Yozgat 66900, Türkiye; 3 Institute for Virology, Düsseldorf University Hospital, Heinrich-Heine-University, 40225 Düsseldorf, Germany; 4 Biotechnology Institute, Ankara University, Ankara 06560, Türkiye; 5 Graduate School of Health Sciences, Ankara University, Ankara 06560, Türkiye; 6 Department of Virology, Faculty of Veterinary Medicine, Hatay Mustafa Kemal University, Hatay 31040, Türkiye; 7 Department of Vaccine Technology, Vaccine Institute, Hacettepe University, Ankara 06100, Türkiye; 8 Republic of Türkiye Ministry of Agriculture and Forestry, Atkaracalar District Directorate, Çankırı 18310, Türkiye; 9 Department of Virology, Faculty of Veterinary Medicine, Kafkas University, Kars 36000, Türkiye; 10 Republic of Türkiye Ministry of Agriculture and Forestry, East Anatolian Agricultural Research Institute, Erzurum 25090, Türkiye; 11 Diagen Biotechnological Systems Health Services and Automation Industry, Ankara 06070, Türkiye; 12 Department of Histology and Embryology, Faculty of Veterinary Medicine, Ankara University, Ankara 06070, Türkiye; 13 Department of Molecular Biology and Genetics, Faculty of Sciences, Karadeniz Technical University, Trabzon 61080, Türkiye; 14 Department of Pathology, Faculty of Veterinary Medicine, Ankara University, Ankara 06070, Türkiye; 15 Department of Biology, Faculty of Sciences, Karadeniz Technical University, Trabzon 61080, Türkiye

**Keywords:** SARS-CoV-2, envelope protein, recombinant, baculovirus

## Abstract

The envelope (env) protein of SARS-CoV-2, a pivotal component of the viral architecture, plays a multifaceted role in viral assembly, replication, pathogenesis, and ion channel activity. These features make it a significant target for understanding virus–host interactions and developing vaccines to combat COVID-19. Recent structural studies provide valuable insights into the conformational dynamics and membrane topology of the SARS-CoV-2 env protein, shedding light on its functional mechanisms. The strong homology and highly conserved structure of the SARS-CoV-2 env protein shape its immunogenicity and functional characteristics. This study examines the ability of the recombinant SARS-CoV-2 env protein to stimulate an immune response. In this study, recombinant envelope proteins were produced using the baculovirus expression system, and their potential efficacy was evaluated in both in vivo and in vitro models. Our results reveal that the env protein of SARS-CoV-2 stimulates humoral and cellular responses and highlight its potential as a promising vaccine candidate for combating the ongoing pandemic.

## 1. Introduction

All scientific communities agree that vaccination is the most effective way to fight the pandemic that has unexpectedly affected the world. Although COVID-19 is no longer a global health emergency, it continues to be a global health threat, particularly with the emergence of new variants. In addition to the use of existing vaccines, the further development of effective and safe new COVID-19 vaccines will be an effective approach in the struggle to counteract new variants.

In general, viruses, including SARS-CoV-2, which causes COVID-19, change over time. Some changes may also affect the properties of the virus, enabling higher transmission ability, altered immune response, a decrease in the performance of vaccines, negative influence on therapeutic drugs and/or diagnostic tools, etc. In this context, some variants have been described as Variants of Concern (VOCs) and *Variants of Interest* (VOIs) for SARS-CoV-2, according to the World Health Organization (https://www.who.int/en/activities/tracking-SARS-CoV-2-variants/, accessed on 15 March 2023).

Coronaviruses (CoVs) have a positive-sense RNA genome and belong to the order Nidovirales. They contain four structural proteins: the spike (S), envelope (E), membrane (M), and nucleocapsid (N) [1]. Our bodies can form antibodies against all structural proteins of the virus [2,3].

The S protein of SARS-CoV-2 plays a critical role in the virus’s entry into cells, and most current COVID-19 vaccines aim to elicit an immune response to this protein. Mutations in the S protein, especially in the receptor binding domain (RBD) region, can increase the virus’s transmissibility and its ability to evade the immune system [4]. According to GISAID data [5], mutations in the S protein are primarily responsible for the emergence of new variants (“CoVariants: SARS-CoV-2 Mutations and Variants of Interest”. https://covariants.org/). In contrast, the E protein of SARS-CoV-2 has a lower mutation rate, with the T9I mutation being the most common. Additionally, M and N proteins have also been studied, revealing a higher mutation rate in the N protein, while the mutation rate in the M protein is similar to that of the E protein (both are low). These findings highlight the need to consider the E protein and other structural proteins as vaccine candidates to combat current and future variants [6,7].

The envelope (env) protein of SARS-CoV, which determines the pathogenicity of the virus, can act as a trigger for a cytokine storm by interacting with the human inflammasome. Therefore, this protein has been selected for the development of active antivirals against SARS-CoV-2 [8]. In addition, it is a small hydrophobic protein with ion channel activity, which has been shown to be highly relevant in virus–host interaction and virulence. The E protein is also an archetype of viroporin. Moreover, this protein has palmitoylation activity, which may regulate its subcellular trafficking and association with lipid rafts [9].

The env proteins of SARS-CoV-2 and SARS-CoV differ only by three substitutions and one deletion in this region [10]. This strong homology makes this protein different from others and provides stability in this domain. It is reported that these conserved domains of the env protein of SARS-CoV-2, as an accessory element, increase viral pathogenicity through protein–protein interaction [8]. In this study, the SARS-CoV-2 env protein was selected because of these aforementioned characteristics. The main purpose of this study is to investigate the potential of the env protein to stimulate the immune system as a vaccine candidate.

## 2. Materials and Methods

### 2.1. Cells and Viruses

Mammalian and insect cell cultures were used in the study. African green monkey kidney (Vero E6, ATCC: CRL-1586) cells were used for all biological assays, including SARS-CoV-2 infectious propagation, infectivity, and virus neutralization assays. Vero E6 cells were cultured in Dulbecco’s modified Eagle’s medium (DMEM) supplemented with 10% Fetal Bovine Serum (FBS) and 1% penicillin/streptomycin at 37 °C under relative humidity. The *Spodoptera frugiperda* (Sf9) cell line used for the expression of recombinant protein was generously provided by Prof. Dr. Monique van Oers from Wageningen University’s Laboratory of Virology. Sf9 insect cells were used for baculovirus cultivation and grown in Sf-900™ II SFM medium (Gibco, Paisley, UK) supplemented with 5% FBS at 28 °C.

The local SARS-CoV-2 isolates, Wuhan-like Ank-1 (MT478018) and Ank-2 (MT478017) [11], and the delta variant (in order to stimulate splenocytes) were used in this study. The SARS-CoV2 Delta variant was isolated from patient nasopharyngeal swabs in September 2021, Ank-Dlt1 (B.1.617.2) (GenBank Acc. No: OM295705). The variant identification was performed after genome-wide sequence analysis following two-step plaque purification for the Delta variant. Viruses were cultured in Vero E6 cells in serum-free DMEM supplemented with L-glutamine, 100 U/mL penicillin, and 100 U/mL streptomycin. Viral infectivity was determined by the Spearman–Kaerber method and expressed as TCID_50_ [12,13]. Briefly, 100 μL of 10-fold dilutions of virus were inoculated in quadruplicate onto Vero E6 cells grown in 96-well plates. Plates were incubated at 37 °C, 5% CO_2_ for at least three days and observed daily for cytopathic effect (CPE).

### 2.2. Construction of Baculovirus Expressing the SARS-CoV-2 Env Protein

The genomic RNA extracted from Wuhan-like Ank-1 isolate was used as a gene source for env protein. The viral RNA was extracted using TRIzol LS reagent (Thermo Fisher Scientific, Rockford, IL, USA) according to manufacturer instructions. The env open reading frame was amplified by reverse transcriptase polymerase chain reaction (RT-PCR) using the following primers: env-F containing *BamHI* site (5′-GGGATCCGCTGATGAGTACGAACTTATG-3′) and env-R containing *PstI* (5′-AACTGCAGGTTCGTTTAGACCAGAAGA-3′). A total of 228 base-pair (bp) PCR products of the env gene were purified and cloned into the pJet 1.2 cloning shuttle vector (Thermo Fisher Scientific, USA) with the TA cloning protocol. The vector was verified by Sanger sequencing and then named pJet-env.

To construct the baculovirus expression cassette for env protein, the Bac-to-Bac™ Baculovirus Expression System (BEV) (Invitrogen, California, USA) was used according to the manufacturer’s instructions. The env gene was excised from the pJet-env vector with *BamHI*/*PstI* enzymes and sub-cloned into the pFactBac HT A (Thermo Fisher Scientific, USA) vector of BEV with the polyhistidine-tags (His-tag). The final vector, pFBHT A-env, was transformed into *Escherichia coli* DH10Bac cells, which harbor an *Autographa californica multiple nucleopolyhedrovirus* (AcMNPV) bacmid DNA. The newly produced recombinant bacmid DNA, including the env gene (AcMNPV-env), was then used for transfection into the Sf9 insect cell line (2 × 10^6^ cells/mL) using a Cellfectin (Invitrogen, USA) transfection reagent. AcMNPV-env was also used to infect Sf9 cells in large volumes to produce the env protein. The infection process was performed at 28 °C. Three days after infection, cells were harvested, and the supernatant containing the AcMNPV-env bacmid was preserved for further protein production in Sf9 cells.

### 2.3. Production and Purification of SARS-CoV-2 Env Protein

AcMNPV-env bacmid was used to re-infect Sf9 cells for large-volume production of env protein. For this purpose, 9 × 10^6^ Sf9 cells grown in T75 flasks were infected with a bacmid solution at a final volume of 0.01 *multiplicity of infection* (MOI). The infected cells were harvested by centrifugation at 800× *g* for 5 min on 4 days post infection (dpi). SARS-CoV-2 env protein fused with the polyhistidine tag was purified by using commercial nickel affinity chromatography following the manufacturer’s instructions (Promega, Madison, WI, USA). As a result of Western blot analysis of the purified env protein, the presence of the protein, expected to be 12 kilodalton (kDa) in weight together with His-tag (3 kDa), was investigated on the membrane. The concentration of the purified protein was determined using Bradford’s method [14].

### 2.4. Animal Experiments

The immunogenicity and protective efficacy of the baculovirus-expressed SARS-CoV-2 env protein were tested in two different strains of animals. The first group consisted of 10–12-week-old Balb/c mice used for immunogenicity experiments, while the second group of animals (same age) were IFNAR^−/−^ used to determine protective efficacy.

In this study, we used three kinds of adjuvants: Freund’s complete/incomplete, CpG (ODN 2395), and alum. Adjuvants enhance immunogenicity and effectiveness, potentially playing active roles in immune response generation and maintenance. As an adjuvant, Freund’s is frequently utilized in animal vaccines, while alum is reliable in human vaccines [15]. CpG, which is a TLR agonist, has also been extensively studied as a vaccine adjuvant, it significantly enhances the CD8^+^ T cell immune response [16]. It is known that CpG is capable of stimulating both cellular and humoral immune responses, and it preferentially induces responses that are Th1-biased [17].

The first group of animals was divided into three subgroups (A, B, and C) of 6 mice each for different formulations (Table 1). On day 0, all animals were exsanguinated and then primed with the respective formulations listed in Table 1. The vaccines were administered intramuscularly. A booster vaccination was performed on day 14 after collecting blood samples from all animals to detect the presence of seroconversion. In order to detect the presence of an immune response, 14 days after the second injection, after blood collection from all groups, 50 µL of a live virus (Wuhan-like strain inoculation dose: 100 TCID_50_: 10^4.75^/0.1 mL) was delivered by the intranasal route. The mouse was restrained by holding it by the scruff in dorsal recumbancy. Then, the challenge virus was inoculated into each nostril using a mechanical pipette (Video S1). After the mouse was returned to its cage, it was observed for any signs of stress. Subsequently, 7 days (on day 35) after the challenge injection, animals in all groups were humanely euthanized. Blood samples and organ tissues were collected for further serological and immunological testing.

Animals in the second group were subdivided into five subgroups (A, B, C, D, E). In this experiment, using different combinations of adjuvants (Freund’s complete and incomplete adjuvant, CpG, and Alum), the recombinant protein (50 µg/mL) was given to 8–10 weeks old IFNAR^−/−^ mice intramuscularly as two injections with a 14-day interval and challenged intranasally (50 µL per nostril) on day 28 with 100 TCID_50_ (10^4.75^/0.1 mL) Wuhan-like local isolate Ank-1. The body weights and temperatures of all the animals were recorded for 7 days post challenge. After sacrificing the animals, the lungs were collected for detection of viral load and SARS-CoV-2 Ank-1 virus infectivity tests by virus titration and RT-qPCR techniques. The spleens were obtained to measure the immune response by flow cytometry. In addition, parts of the tissues were taken for histopathological and immunohistochemical examinations.

### 2.5. Evaluation of SARS-CoV-2 Anti-Env Antibodies by Indirect ELISA

The presence of SARS-CoV-2 env-specific antibodies in the mouse serum samples was determined by indirect ELISA. Some 96-well plates (Greiner GmBh, Pleidelsheim, Germany) were coated (5 μg/well) with baculovirus-expressed env protein in phosphate-buffered saline (PBS) pH 7.2 at 4 °C overnight. After washing with PBS-T (PBS + 0.05% Tween-20), the plates were blocked with PBS-T containing 1% bovine serum albumin (BSA) for 1 h at room temperature. The plates were washed once again with PBS-T and then incubated with serial dilutions (from 1/50 to 1/6400) of mouse sera (100 μL/well) for 2 h at room temperature. After three washes, as described above, horseradish peroxidase (HRP)-conjugated goat anti-mouse IgG antibody (1:10,000) was added to each test well and incubated for 1 h at room temperature. The plates were washed three times with PBS-T, and 100 µL of 3,3′,5,5′-tetramethylbenzidine (TMB) substrate solution (Thermo Fisher Scientific, USA) was added to each well and incubated for a further 20 min at room temperature in the dark. The reaction was stopped with the addition of 3M H_2_SO_4_, and absorbance was measured immediately at 450 nm using an ELISA reader.

### 2.6. Determining Neutralizing Antibodies against SARS-CoV-2 via the VNT

The presence of SARS-CoV-2-neutralizing antibodies in serum samples from animals was tested by the VNT. Serum samples were heat-inactivated at 56 °C for 30 min. Sera were serially diluted two-fold, starting at 1/5, in DMEM supplemented with 1% penicillin–streptomycin. Then, 50 µL of serial dilutions of each serum were mixed with 50 µL of 100 TCID_50_ of SARS-CoV-2 in the 96-well tissue culture plate, and each serum dilution was tested in ten replicates. After incubation for 1 h at 37 °C in 5% CO_2_, 50 µL of Vero cells were added at a concentration of 3 × 10^5^ cells/well. Titer confirmation of the test virus was made by diluting 10-fold using the back titration method. The plates were further incubated at 37 °C for 4 days. Cells were examined for CPE by inverted light microscopy. Neutralization titers were expressed as the reciprocal of the highest dilution of serum that resulted in at least a 50% reduction in the number of infected cells relative to the negative control.

### 2.7. Determining Viral Load in the Lungs by RT-qPCR

After euthanasia of Group 2 mice, lung tissues were stored in RNAlater™ stabilization solution (Thermo Fisher Scientific, USA) at −86 °C. Frozen lung tissues were homogenized using a tissue homogenizer at 10,000 rpm for 15 s. Total RNA was isolated using PureZOL™ RNA Isolation Reagent (Bio-Rad, California, USA) according to the manufacturer’s instructions. Total RNA concentrations were measured using a NanoDrop™ One Microvolume UV–Vis spectrophotometer (Thermo Fisher Scientific, USA), and integrity was checked via agarose gel electrophoresis.

A total of 1 µg of RNA samples were used to detect the SARS-CoV-2 viral load using the Luna^®^ Universal One-Step RT-qPCR Kit (NEB, Massachusetts, USA). The reaction mix was prepared according to the manufacturer’s instructions. SARS-CoV-2 viral RNA was amplified using the following primer and probe combination targeting the S protein coding region: 

Forward primer (5′-GATCTCTGCTTTACTTAATGTCTATGC-3′); 

Reverse primer (5′-CGCAGCCTGTAAAATCATCTG-3′); 

Probe (5′ FAM-TTGCCCTGGAGCGATTTGTCTGA BHQ1-3′).

### 2.8. Detection of Cytokine Levels by Flow Cytometry

In this study, the cytokine responses in the virus-stimulated splenocytes of immunized IFNAR^−/−^ mice were analyzed. Mouse splenocytes were aseptically collected from all the immunized IFNAR^−/−^ mice. Then, red blood cells (RBCs) were lyzed in RBC lysis buffer (Thermo Fisher, USA) and washed with PBS. In the next step, the cell pellets were resuspended in DMEM, and separated mouse splenocytes (1 × 10^6^ cells/mL) were cultured. Then, mouse splenocytes were stimulated with the SARS-CoV-2 Delta variant. Supernatants were collected after 24 and 48 h and assayed with the LEGENDplex^TM^ Mouse Th Cytokine Panel kit (BioLegend, San Diego, CA, USA). The test was performed on a V-bottom plate according to the manufacturer’s instructions. The results were obtained by using CytoFlex cytometry on the day of the assay, and the data were analyzed using Cytexpert software (Beckman Coulter, Brea, CA, USA). Statistically, a two-way ANOVA and Tukey’s multiple comparison test were used to compare the differences in the cytokine levels. All figures were generated using GraphPad Prism 9.0 (GraphPad Software).

### 2.9. Immuno-Histochemical Staining

The Avidin Biotin Complex Peroxidase method was used by modifying the kit procedure. The tissue samples (lung, liver, kidney, and brain) were fixed in 10% neutral buffered formalin and processed and paraffin-blocked for histology using standard methods. Afterwards, we carried out deparaffinization and rehydration of tissue sections taken from paraffin blocks prepared for histopathological examinations to adhesive (APES) slides in a 3% hydrogen peroxide–methanol mixture (20 min) to prevent endogenous peroxidase activity at room temperature; we did so using trypsin (12 min) and EDTA buffer (25 min at 100 °C). They were incubated in protein-blocking serum for 10 min at 37 °C. Then, the primary antibody (1:1000 dilution) was applied (1 h) in the humidity chamber. The incubation was carried out with a biotininized antibody and streptavidin peroxidase for 15 min at 37 °C. Afterwards, the sections were incubated in 3-amino-9-ethylcarbazole (AEC) solution and Mayer’s hematoxylin (3–5 min), washed in running tap water, and covered with gel. The sections were washed with PBS between all steps indicated, with the exception of the protein-blocking step.

Upon imaging, the staining in the target cells was indicated to be strong or weak in the 40× objective lens, according to the diffuse and intense staining feature of the immunopositivity. All staining was evaluated under a light microscope (Leica DM4000) and photographed (Leica DF-280) (Leica Microsystems Inc., North Deerfield, IL, USA).

Detected positive cells were graded semi-quantitatively according to their coverage and intensity in ten different microscope fields at 40× magnification according to the criteria specified below:

(−) Immunopositivity is not detected;

(+) Detection of less than 10% immunopositivity (mild);

(++) Detection of 10–50% immunopositivity (moderate);

(+++) Detection of more than 50% strong immunopositivity (severe).

## 3. Results

### 3.1. Protein Expression and Determination of Protein Concentration

**Figure 1:**
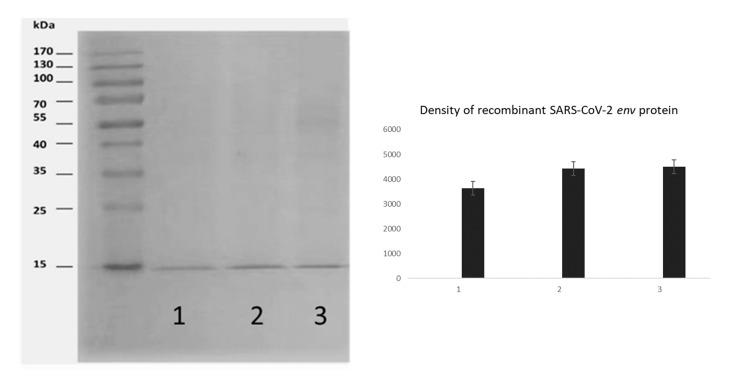
Western blot analysis of the env protein produced in the baculovirus vector system. The intensity of the protein bands was quantified using densitometry analysis performed with ImageJ software (Version 1.54j). The resulting intensity ratios are shown beneath each band, providing a comparative measurement of protein expression levels.

The SARS-CoV-2 envelope gene was expressed in the baculovirus vector system. The constructed bacmid *AcMNPV* and env gene (*AcMNPV*-env) were used to infect Sf9 cells, and the env protein was produced at 125 µg/mL concentration. Figure 1 shows the Western blot analysis of the purified recombinant env protein, which has a molecular weight of 12 kDa with a His-tag (3 kDa). The purified env protein was used in the following experiments.

### 3.2. Challenged Animals

In the first animal experiment, three groups of 8–10 weeks old Balb/c mice were formed. The body temperatures and weights of the animals in all were monitored before and after the challenge virus administration, and they showed no significant difference among groups of mice inoculated with the recombinant env protein with Freund’s complete and incomplete adjuvant, or PBS. In addition, no clinical signs were observed during the 28-day period before giving the challenge virus. The second animal experiment was performed to understand the efficacy of different adjuvants with six groups of 8–10-week-old IFNAR^−/−^ (knockout for type I interferon receptors) mice. Similar results were also obtained in this experiment; no clinical signs were observed during the 28-day period before the challenge virus was given.

### 3.3. Determination of Antibodies by Indirect ELISA and VNT

Blood samples were taken 14 days after the second immunization for the determination of antibodies by indirect ELISA for both experiments. The results of the ELISA revealed that both animal groups immunized with env protein seroconverted against SARS-CoV-2. Accordingly, strong antibody positivity was detected at the 1/3200 dilution step in the sera of mice vaccinated with the recombinant protein. A positivity rate of 1/6400 was also observed in some sera.

The results of the second experiment, which was performed to understand the efficacy of different adjuvants, showed that the highest antibody response was detected at the 1/6400 serum dilution in the animals immunized with Freund’s complete and incomplete adjuvants, while in the other adjuvant groups’ antibody responses were detected in the range of 1/1600–1/3200 serum dilutions.

The results of the virus neutralization test (VNT) determined that the SARS-CoV-2 Ank-1 env recombinant protein stimulated an immune response in Balb/c mice and induced the formation of neutralizing antibodies at rates ranging from 1/60 to 1/120. In the second experiment, which was performed to understand the efficacy of different adjuvants, similar results were detected. The lowest neutralizing antibody rate (1/20 and 1/30) was detected in IFNAR^−/−^ mice (n = 2) that were inoculated with the env protein with a CpG + Alum adjuvant combination. The highest neutralizing antibody rate (1/60) was found in the complete incomplete adjuvant group, with a similar result in the first experiment. In the negative control groups given only adjuvant, no neutralizing antibody response was detected, as expected.

### 3.4. Viral Load Determination in Lung Samples by RT-qPCR and Virus Titration

The viral loads were measured in lung tissue from all groups by RT-qPCR. Accordingly, only the positive control group and adjuvant-injected groups were detected as positive. Additionally, the Alum + recombinant env protein-injected group was found to be moderately positive. No positivity was detected in the lung samples of the mice that were given the challenge virus after vaccination with recombinant protein.

### 3.5. Immunoperoxidase Staining

**Figure 2:**
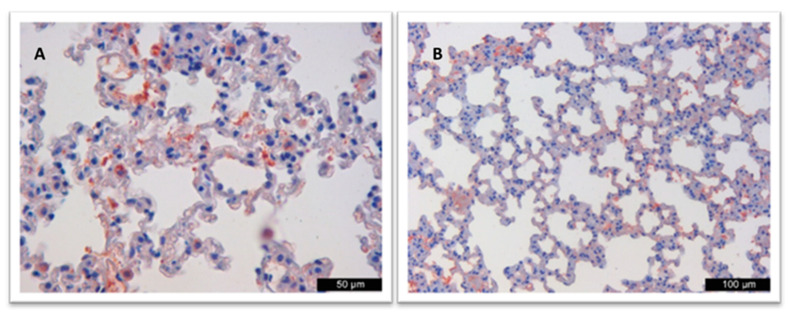
(**A**) Immunopositivity in the alveolar epithelial cells and macrophages of lungs. (**B**) Immunonegative lung.

In the examined lung samples of the positive control group, especially in the alveolar epithelial cells and macrophages, moderate-intensity (++) immunopositivity was observed in the cytoplasm, as indicated by mostly homogeneous brownish staining, while antigen was not detected in the liver, kidney, and brain samples. In addition to this, antigen positivity was not detected in the lung, liver, kidney, and brain samples of the negative control group (Figure 2).

**Figure 3:**
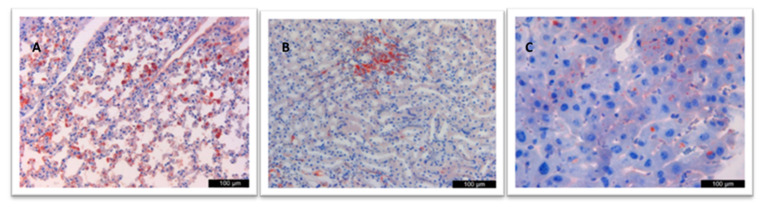
Immunopositivity in the (**A**) lung, (**B**) kidney, and (**C**) liver.

In the examined lung samples of groups vaccinated with only adjuvant (without protein), especially in the alveolar epithelial cells and macrophage cytoplasm, immunopositivity in the form of severe (+++) granular and homogeneous brown staining was noted. At the same time, there were interstitial inflammatory cells in the cytoplasm of both kidneys, and similar immunopositivity was observed in the cytoplasm of hepatocytes in the liver (Figure 3).

**Figure 4:**
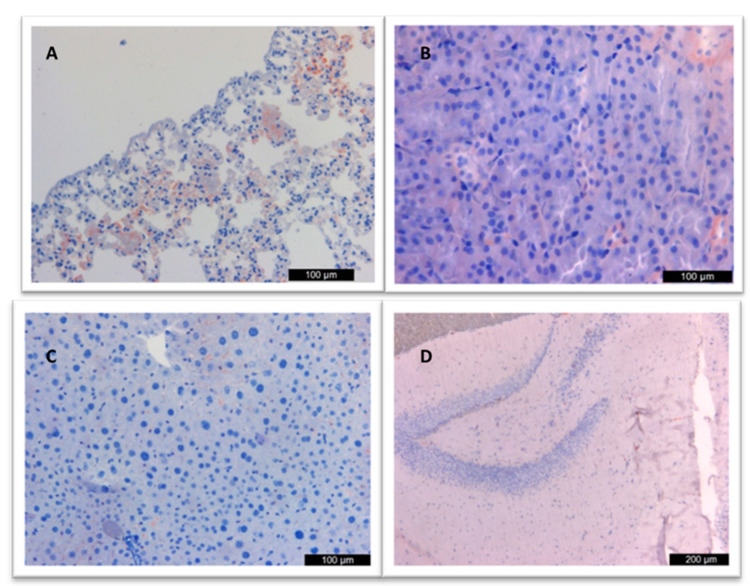
(**A**) Immunopositivity in alveolar epithelial cells of the lung, (**B**) Immunonegativity in the kidney, (**C**) liver, and (**D**) cornu ammonis.

In the examined lung samples of the vaccinated group with recombinant env protein+adjuvant, mild (+) granular and homogeneous brown staining and immunopositivity were noted, especially in the cytoplasm of bronchiolar epithelial, perivascular, and peribronchiolar inflammatory cells. Similar staining was observed on the bronchiolar epithelial surface. Antigen was not detected in liver, kidney, and brain samples (Figure 4).

### 3.6. Determination of Infection Blockage in Spleen Samples of Mice Vaccinated with Env Protein

According to the results of back titration, which was performed with virus-stimulated spleen samples of mice, the amount of virus in the splenocytes of the mice vaccinated with env protein was lower than that of the group given only adjuvant and PBS.

In experimental groups receiving recombinant protein vaccination, when the results of RT-qPCR and virus titration experiments in the lungs were evaluated, the presence of mild viral infection in the lungs was found in complete and incomplete adjuvant combinations and only alum adjuvant applications. Moreover, considering the ct values, it seemed that these infections were close to ending. At the same time, there was a similar situation in the virus titration results from the lungs. In parallel with the real-time and virus titration results, there were signs of mild infection in immunohistochemical positivity in the lungs.

In the experimental groups in which CpG and CpG + Alum adjuvants were used together with recombinant protein, no positivity was detected in RT-qPCR and virus titration tests in the lungs.

### 3.7. Detection of Cytokine Levels

SARS-CoV-2 Ank-1 isolate recombinant env protein-specific T cell responses were evaluated by measuring the levels of Th1 cytokines (IFN-γ, IL-2, TNF-α) following the stimulation of splenocytes with the challenge (delta) virus. Th2 cell responses were evaluated by IL-4, IL-5, IL-6, IL-10, and IL-13 concentration measurements. 

**Figure 5:**
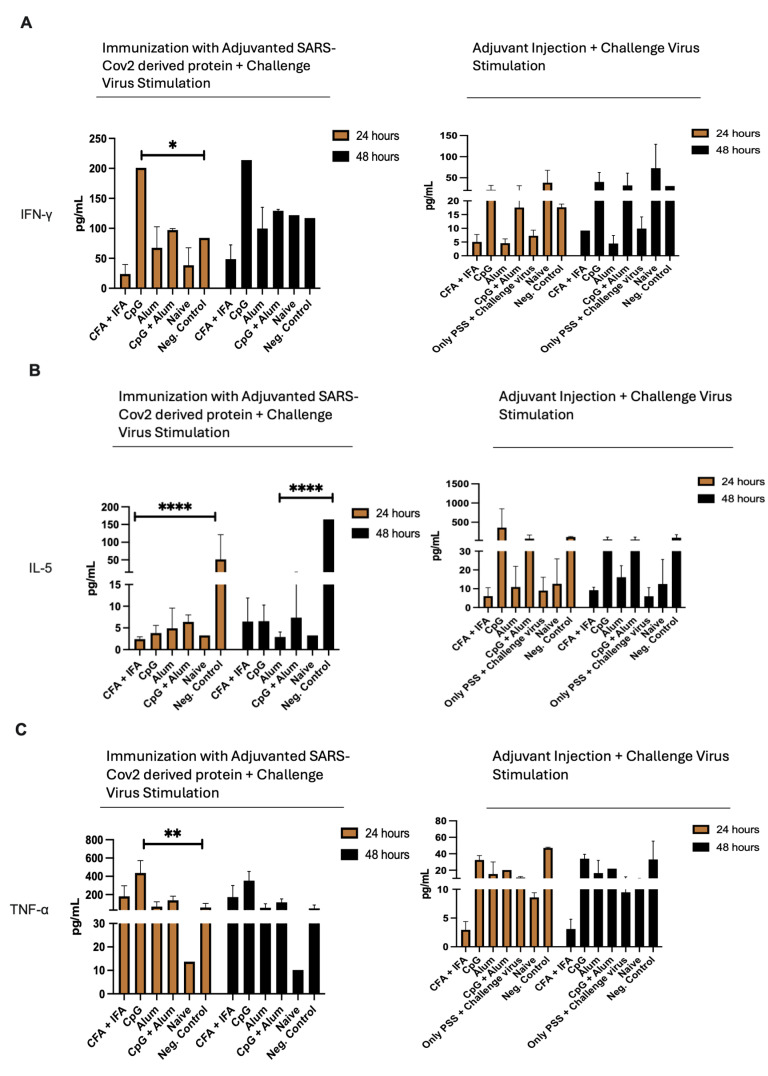

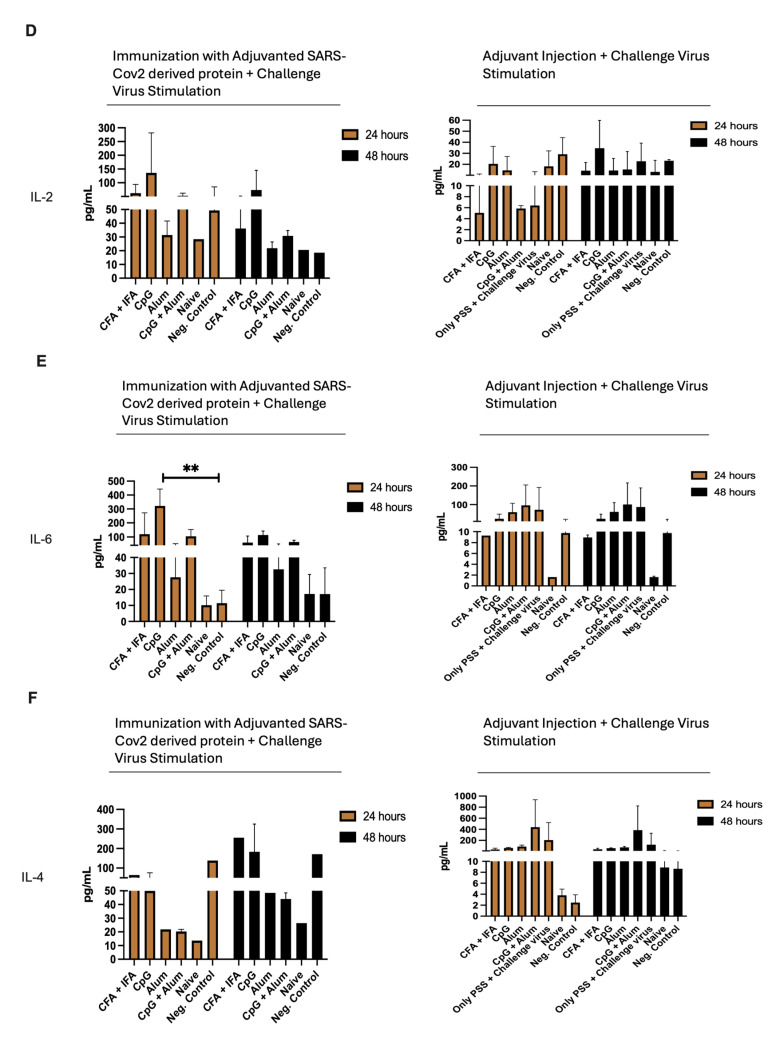

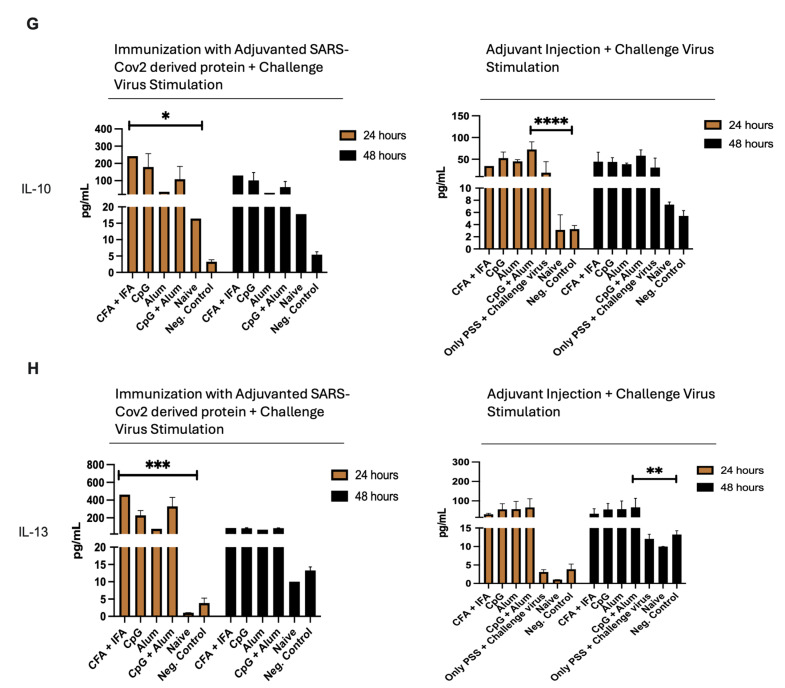
(**A**–**H**). Graphs on the left show representative results of cytokine responses upon SARS-CoV-2 Ank-1 recombinant env protein immunization with different adjuvants at 24- and 48 h after challenge virus stimulation. Graphs on the part show the cytokine concentration levels after only adjuvant injection and challenge virus stimulation as a positive control. (**A**) IFN-γ. (**B**) IL-5. (**C**) TNF-α. (**D**) IL-2. (**E**) IL-6. (**F**) IL-4. (**G**) IL-10. (**H**) IL-13. Comparisons were made via a two-way ANOVA with Tukey’s multiple comparisons test to determine statistical significance. **** *p* < 0.0001, *** *p* < 0.001, ** *p* < 0.01, * *p* < 0.05.

Regarding IFN-γ titers, the CpG + env protein triggers IFN-γ release within 24 h upon challenge virus stimulation compared to naïve and negative control mice (Figure 5A). Our results indicate that immunization with env protein leads to higher levels of TNF-α production (Figure 5C). It can be concluded that CpG adjuvanted vaccination with env protein induces stronger Th1 responses against SARS-CoV-2. Our findings show that immunization, especially CpG-env protein, slightly increases IL-2 levels within 24 h compared to naïve and negative control mice. Additionally, mice that received only different adjuvants and were challenged with SARS-CoV-2 showed lower levels of IL-2 compared to the mice immunized with env protein (Figure 5D).

Immunization with env protein led to lower levels of IL-5 production within 24- and 48 h compared to the mice group, which was injected only with adjuvant (Figure 5B). Our results show that immunization, especially in the CpG-env group, led to higher IL-6 levels within 24 h compared to negative control mice. Interestingly, Alum-adjuvanted immunization caused no significant difference from the negative control. Mice that received only adjuvant and were challenged with SARS-CoV-2 showed generally slightly lower levels of IL-6 (Figure 5E). According to our results, immunization with especially Alum and CpG + Alum-adjuvanted env protein caused, within 24 h, lower IL-4 levels compared to the negative control mouse. This situation remained for 48 h. Furthermore, the mice group that received only CpG + Alum adjuvant or PBS and was challenged with SARS-CoV-2 showed significantly higher levels of IL-4 compared to immunized mice (Figure 5F). Our findings demonstrate that the env protein-immunized mice showed significantly higher IL-10 levels compared to negative control mice upon 24- and 48 h challenge virus stimulation (Figure 5G). Interestingly, our results show that immunization with env resulted in higher levels of IL-13 production (Figure 5H), whereas in the immunized mouse group, IL-4 production was reduced compared with mice who received only adjuvant and the negative control group.

Representative figures demonstrate IFN-γ, IL-5, TNF-α, IL-2, IL-6, IL-4, IL-10, and IL-13 production from splenocytes after their stimulation with SARS-CoV-2 env protein after 24 and 48 h (Figure 5). The statistical significance of the experimental results is presented in Table 2.

## 4. Discussion

The emergence of the SARS-CoV-2 infection has caused devastating health problems and economic loads worldwide since 2019. In this context, vaccination is considered the ideal way to safely achieve herd immunity against COVID-19 infection. With this aim in mind, we planned in this study to design a novel vaccine candidate to fight against the SARS-CoV-2 infection. The env protein is known as the smallest outer surface protein of the SARS-CoV-2 genome, harboring B cell and T cell epitopes with the potential to produce a high immune response [18]. Based on these features, the env protein was exploited as a vaccine candidate against SARS-CoV-2 infection in this study.

The baculovirus gene expression system, having high transgene capacity, enables both easy construction of recombinant viruses with a bacmid system and post-translational modification with a eukaryotic system such as glycosylation. In addition, another important advantage is its replication defect, i.e., the absence of a primary antibody and low cytotoxicity compared to mammalian virus-derived vectors [19]. It is known that several CpG motifs are present throughout baculoviral DNA and induce an antiviral response in mammalian cells. Therefore, as an expression vector system, we preferred baculovirus in this study.

There are limited studies evaluating the immunoprotective potential of the SARS-CoVs env protein as a candidate vaccine [18,20,21]. One of the reasons for this is that the S protein plays a critical role in the virus’s entry into cells and is a primary target in vaccine development due to its capacity to elicit antibody responses [22]. The high mutation rate of the S protein leads to the emergence of variants, necessitating the tracking and targeting of these mutations. Variant mutations primarily occur in the spike protein, while the E protein has remained highly conserved in *Variants of Concern* (VOCs).

The SARS-CoV-2 E protein was identified as a potential target for vaccine development even before the emergence of variants [23]. In a docking-based study, the structural evolution and conformational changes of the E protein were analyzed, revealing that it interacts better with immune cells and exhibits strong binding affinity with human leukocyte antigens (HLAs) [18]. Additionally, it was found that the E protein is suitable for recombinant production and presents a promising target for future subunit vaccine development. In an in silico approach aimed at developing a chimeric peptide E protein-based COVID-19 vaccine, it was shown that the vaccine has a high affinity for major histocompatibility complex (MHC) molecules [24].

In our study, the data showed that considerable cellular immune responses as well as humoral immunity were elicited in IFNAR^−/−^ mice. The antibody responses against SARS-CoV-2 were investigated in mice immunized with recombinant env protein expressed in baculovirus. All mice immunized two times with a 14-day interval of recombinant SARS-CoV-2 env protein developed neutralizing antibody titers of >1:20. Additionally, IgG titers reaching 1:6400 have been detected by using homemade ELISA. Obtaining specific humoral response results indicated that recombinant baculoviruses expressing SARS-CoV-2 env protein could provide protective immunity against the SARS-CoV-2 challenge.

Studies demonstrate that the survival of SARS-CoV-2-infected patients is contingent on both cell-mediated immune responses (Th) and neutralizing antibodies [25]. Our results revealed that vaccination with the recombinant env protein triggered the cellular immune response. It is known that the distinct subsets of Th cells, such as Th1 (IL-2, IFN-γ, and TNF-β) and Th2 (IL-4, IL-5, IL-6, IL-10, and IL-13), can be determined by cytokine secretion upon activation. Natural killer (NK) cells and CD8^+^ T cells mainly produce IFN-γ. This cytokine shows immunostimulant effects and plays significant roles in the restriction of viral infection [26]. TNF-α is predominantly secreted by activated macrophages, T lymphocytes, and NK cells, and is highly associated with inflammation [27]. IL-2 is generally produced by activated CD4^+^ and CD8^+^ T cells during a primary immune response to facilitate differentiation of Th precursors into immune-effective cells [28,29]. It was found in our study that vaccination by using CpG adjuvant with SARS-CoV-2 Ank-1 recombinant env protein induces high Th1 responses against SARS-CoV-2. Our findings are supported by a high-level production of Th1 cytokines, especially IFN-γ, in the CpG adjuvanted immunized mouse group compared to naïve and negative control mice. CpG-adjuvanted immunizations with recombinant env protein indicated that high levels of IFN-γ, IL-2, and TNF-α trigger Th1 immune responses within 24 and 48 h. The higher levels of the IL-2 upon stimulation with the challenge virus in the mouse group immunized with env protein + CpG showed that the SARS-CoV-2 envelope protein induced stronger Th1 cell responses. This situation contributed to higher cytokine concentration levels in immunized mouse groups regarding IFN-γ and TNF-α.

Th2 responses affect COVID-19 prognoses negatively due to high levels of Th2 cytokines. IL-4 regulates the differentiation of naïve CD4^+^ T cells into effector Th2 cells [30]. It has been shown that SARS-CoV-2 infection causes higher levels of IL-4 production in infected patients [31]. IL-5 is a key cytokine in the regulation of Th2 immune responses and is highly associated with eosinophil activation. It has been reported in some studies that patients with severe COVID-19 have increased IL-5 levels [32]. According to our results, immunization with env protein may help to reduce lung damage when encountering SARS-CoV-2. This idea was supported by the higher IL-5 levels in negative control mice, which were stimulated with the challenge virus without any protein or adjuvant application. In this context, immunization with env protein contributed to keeping IL-5 and IL-4 levels stable for 24- and 48 h upon challenge virus (Delta) stimulation.

IL-6 is predominantly produced by macrophages and can act as a proinflammatory cytokine during innate immune responses [33,34]. IL-10 inhibits effective immune responses, and it has been classified as an anti-inflammatory cytokine [35]. It has been reported that IL-10^−/−^ mice show higher levels of virus clearance and better survival rates during acute influenza A virus infection than wild-type mice [36]. Interestingly, it has been demonstrated that elevated IL-10 levels positively correlate with the severity of COVID-19, especially in patients with diabetes [37]. However, IL-10 may play a dual role during COVID-19 infection [38]. Low IL-10 levels may contribute to the resolution of COVID-19 infection but possibly increase the magnitude of lung damage via strong early immune responses. In this context, our findings concluded that immunization with SARS-CoV-2 env protein may help to protect lung tissue from strong early immune-mediated damage.

IL-13 is produced by Th2 cells, natural killer T cells, eosinophils, and basophils. In particular, the secondary structures of IL-4 and IL-13 have much in common [39]. According to our findings, high IL-13 levels have been shown in the mouse groups immunized with recombinant env protein. These results show the presence of a strong Th2 immune response in immunized mice.

In this study, three different types of adjuvants were used: Freund’s adjuvant, CpG and Alum. We found that vaccination using a CpG adjuvant with SARS-CoV-2 Ank-1 env protein induces stronger Th1 responses than the other adjuvants. However, no significant difference was detected among the used adjuvants in neutralizing antibody stimulation.

Although serological tests are mostly used in producing vaccines against many viral agents, including SARS-CoV-2, the immunohistochemical detection of antigen produces important results in terms of determining efficacy and sensitivity [40]. However, non-specific or false positives have been observed, especially in parenchymatous organs (brain, lung, kidney, etc.). Therefore, modifications to the method of tissue staining should definitely be made.

At the same time, the type of immunological response to the stimulus of the protein types used is also very important [21]. However, in the study, these features were not examined, and the presence of antigen was evaluated semi-quantitatively. Significant differences were noted between immunized animals and non-immunized animals.

RT-qPCR, virus titration assays, and immunohistochemistry results in the lungs show very low virus presence in these tissues. The results of this study indicate that the recombinant envelope protein obtained from the SARS-CoV-2 Ank-1 strain can stimulate both humoral and cellular immune responses that are essential for controlling the dissemination of infection and to inhibiting the entry of the virus.

This study presents several limitations that should be considered when interpreting its results. First, although the animal models used to assess the immune response have demonstrated the efficacy of the SARS-CoV-2 envelope protein, they may not fully mimic the complex interactions and immune environment in humans. Therefore, further studies in models closer to humans, such as non-human primates, may provide more reliable data on the expected efficacy and safety of this vaccine candidate in humans. Secondly, although this study provided evidence that the SARS-CoV-2 env protein has immunogenic properties, the number of samples used in both in vivo and in vitro experiments was limited in accordance with ethical principles. Additionally, the durability and change of immune response over time in vaccinated individuals were not examined. In this context, long-term studies are needed to evaluate the persistence of vaccine candidate-derived antibodies and memory cells. Considering the constantly evolving structure of SARS-CoV-2, we believe that the viral envelope protein should be included in next-generation vaccines so that we are prepared for the future; this protein should be used to develop effective strategies for fighting current and future variants.

## 5. Conclusions

In this study, we demonstrated the potential of using the SARS-CoV-2 envelope protein as a protective vaccine candidate against infection. The highly conserved nature and strong homology of the envelope protein contribute to its immunogenicity and functional properties. Considering the continuously evolving nature of the virus, the broad-spectrum potential of the envelope protein positions it as a significant component in the development of next-generation vaccines. This study provides a strategic advantage in combating current and emerging virus variants related to COVID-19 infection, contributing to efforts to develop a universal vaccine antigen.

## 6. Patents

A patent application has been filed for this recombinant protein (application No.: 2023/008078, Application Date: 11.07.2023, Invention Title: SARS CORONAVIRUS-2 ENVELOPE PROTEIN PRODUCED IN BACULOVIRUS EXPRESSION SYSTEM. Reference No.: 2289P02).

## Figures and Tables

**Table 1:** Animal strain subgroup and vaccination information of the experimental groups included in this study.

Groups	Animal Strain	Subgroups		Vaccine Formulation	Timing			
					Day 0	Day 14	Day 28	Day 35
1	Balb/c	A		*env* protein + Freund’s adjuvants	Priming	Boosting	Challenged	Sacrificed
		B		Freund’s adjuvants	Priming	Boosting	Challenged	Sacrificed
		C	Negative control	-	PBS	PBS	PBS	Sacrificed
			Positive control	-	PBS	PBS	Challenged	Sacrificed
2	IFNAR^−/−^	A		*env* protein + Freund’s adjuvants	Priming	Boosting	Challenged	Sacrificed
		B		*env* protein + CpG adjuvant	Priming	Boosting	Challenged	Sacrificed
		C		*env* protein + Alum adjuvant	Priming	Boosting	Challenged	Sacrificed
		D		*env* protein + CpG + Alum adjuvant	Priming	Boosting	Challenged	Sacrificed
		E	1	Freund’s adjuvants	Priming	Boosting	Challenged	Sacrificed
			2	Alum adjuvant	Priming	Boosting	Challenged	Sacrificed
			3	CpG adjuvant	Priming	Boosting	Challenged	Sacrificed
			4	CpG + Alum adjuvant	Priming	Boosting	Challenged	Sacrificed
			Negative control	-	PBS	PBS	PBS	Sacrificed
			Positive control	-	PBS	PBS	Challenged	Sacrificed

**Table 2:** *p*-Values of Th1 and Th2 cytokine groups.

Cytokine Group	Cytokine	*p*-Value	Statistical Significance
	IFN-γ	<0.05	*
Th1	IL-2	>0.05	ns
	TNF-α	<0.01	**
	IL-4	>0.05	ns
	IL-5	<0.0001	****
Th2	IL-6	<0.01	**
	IL-10	<0.05	*
	IL-13	<0.001	***

ns: non-significant differences, **** *p* < 0.0001, *** *p* < 0.001, ** *p* < 0.01, * *p* < 0.05.

## Data Availability

The data that support the findings of this study are available from the corresponding author upon reasonable request.

## Supplementary Materials

The following supporting information can be downloaded at: https://www.mdpi.com/article/10.3390/vaccines12090977/s1, Video S1: Challenge virus inoculation via intranasal route.
